# Exploring the Fundamental Dynamics of Error-Based Motor Learning Using a Stationary Predictive-Saccade Task

**DOI:** 10.1371/journal.pone.0025225

**Published:** 2011-09-23

**Authors:** Aaron L. Wong, Mark Shelhamer

**Affiliations:** 1 Department of Biomedical Engineering, The Johns Hopkins University, School of Medicine, Baltimore, Maryland, United States of America; 2 Department of Otolaryngology – Head and Neck Surgery, The Johns Hopkins University, School of Medicine, Baltimore, Maryland, United States of America; The University of Western Ontario, Canada

## Abstract

The maintenance of movement accuracy uses prior performance errors to correct future motor plans; this motor-learning process ensures that movements remain quick and accurate. The control of predictive saccades, in which anticipatory movements are made to future targets before visual stimulus information becomes available, serves as an ideal paradigm to analyze how the motor system utilizes prior errors to drive movements to a desired goal. Predictive saccades constitute a stationary process (the mean and to a rough approximation the variability of the data do not vary over time, unlike a typical motor adaptation paradigm). This enables us to study inter-trial correlations, both on a trial-by-trial basis and across long blocks of trials. Saccade errors are found to be corrected on a trial-by-trial basis in a direction-specific manner (the next saccade made in the same direction will reflect a correction for errors made on the current saccade). Additionally, there is evidence for a second, modulating process that exhibits long memory. That is, performance information, as measured via inter-trial correlations, is strongly retained across a large number of saccades (about 100 trials). Together, this evidence indicates that the dynamics of motor learning exhibit complexities that must be carefully considered, as they cannot be fully described with current state-space (ARMA) modeling efforts.

## Introduction

Motor learning is the mechanism by which neural control processes are updated – typically via observation of and compensation for errors – to keep actions quick and accurate. Motor adaptation handles the latter: as the environment and the body change over time, the motor controller adapts movement gains in response to prior error information. This keeps movements accurate despite short-term effects such as fatigue and long-term changes such as muscle atrophy resulting from aging. To address the desire for rapid movements, which are hindered by feedback delays, the brain can generate predictive behaviors that anticipate the motor action to be performed. This reduces the time required to respond to a stimulus, allowing targeted movements such as swinging a bat to hit an oncoming baseball to be executed successfully. To keep the gain of such anticipatory movements accurate, the motor system relies upon previous prediction errors to modulate future behavior – the same error detection and processing that drives motor adaptation [1]. Thus, *predictive* actions that produce consistent responses rely upon the same motor learning mechanisms that drive *adaptation*.

One sensorimotor task that utilizes motor learning to maintain performance is the control of predictive-saccade amplitudes. This task consists of making periodically-paced saccades (rapid eye movements) between two alternating targets at fixed locations. After a few trials, saccades automatically and involuntarily become anticipatory: they are initiated with latencies of 70 msec or less, as opposed to typical reactive saccades that begin 250 msec after target onset [2]−[5]. Since it takes nearly 70 msec for visual information to reach cortex, it is unlikely that these movements are visually guided; hence, they must be planned in advance of target onset. In fact, predictive saccades may have latencies as low as −200 msec, meaning they are completed well before the visual target appears. These features make predictive saccades distinct from other saccade types, including express saccades that also have shorter latencies than reactive saccades but are still visually guided, or memory-guided saccades that must be intentionally generated as an active recall of a prompted location [6]−[7]. Furthermore, unlike saccades made repeatedly in the dark to remembered targets with no visual feedback – which become increasingly inaccurate with repetition [8] – predictive saccades remain reasonably accurate for hundreds of trials. This suggests the presence of an active motor-learning process.

Spatial performance on this task can be considered statistically to be first-order stationary, in the sense that the goal of the task is to maintain a constant-sized movement throughout the paradigm. In other words, the average saccadic amplitude remains constant. This provides a distinct advantage over typical motor adaptation tasks, which request a change in movement gain in response to artificially exaggerated errors. In such cases, while the effect of learning is quite apparent, it becomes difficult to separate the nonstationary change in gain – part of which results from a cognitive strategy to counteract observed errors [9]−[12] – from the underlying learning dynamics. While many attempts have been made to classify the system properties involved in adaptive processes, they suffer the drawback of being primarily concerned with the nonstationarity and ignore information contained within variability about this adaptive trend. Adaptation is often analyzed with static state-space models (equivalently, Auto-Regressive Moving-Average, or ARMA, models) fit to the observed gain change (i.e., the nonstationary trend) [13]−[15]. However, these time-series analysis methods were developed to describe the dynamics of *stationary* behaviors, and typically suggest methods for removing trends as the first step of model fitting (for example, see [16]). When state-space (ARMA) models (which assume that statistics including the mean of the time series do not vary across time) are applied to nonstationary data (i.e., an adaptive change of movement gain), model parameters should theoretically be time-dependent. Problems arising from the use of time-invariant models to fit adaptation data have been previously suggested in the literature. Zarahn et al. demonstrated that single state-space (ARMA) models cannot explain the phenomenon of savings [17], in which learning is faster during a repeated exposure to the adaptive stimulus following a washout period between sessions. Instead, these authors found that the data were better fit with a model whose parameters varied in each phase of the experiment (learning, washout, and relearning), which is exactly what would be expected when fitting nonstationary data with a stationary model.

**Figure 1:**
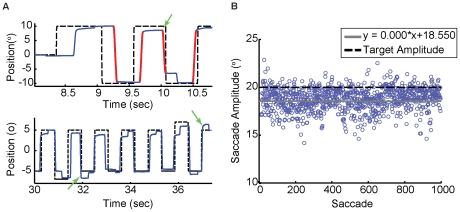
Experimental paradigm and sample data. A: Eye movements (blue line) recorded as one subject made saccades (red highlighted sections of the eye-movement trace) to a periodically moving target (black dashed line). Within a few saccades, subjects automatically began anticipating the future target location, and saccade latency decreased below 70 msec (arrow, upper panel). The lower trace demonstrates what occurs when catch trials are interspersed throughout the paradigm. After observing unexpected catch trials, subjects greatly increase their saccade gain on the next trials in response to the unusually large errors (arrows, lower panel). B: Despite generating a large number of predictive saccades (1000, in this case), subject performance appears weakly stationary (constant mean and, to a close approximation, constant variance).

Aside from this potential pitfall, state-space (ARMA) models also imply that inter-trial correlations decay at an exponential rate. Such rapid decay of information across trials, however, has been previously demonstrated to be insufficient to describe some classic features of motor learning. Studies in rhythmic finger tapping [18]−[19] and in the temporal control of predictive saccade *latencies* [5], [20] suggest that there may be retention of information across trials that are greatly separated in time, beyond that which can be readily described by a simple state-space (ARMA) model. Instead, such processes may exhibit statistical *long memory* (long-range dependence), in which inter-trial correlations decay as a power law. By examining a first-order stationary process such as the control of predictive saccades (which, to a rough approximation, can be considered weakly stationary since the variability about the mean also appears essentially time-independent; see Figure 1B), it is possible to properly apply time-series analysis techniques to examine and describe the dynamics of motor learning.

In this study, we use a careful time-dependent computational approach to explore the processes that underlie motor learning by studying the control of predictive-saccade amplitudes. We show evidence consistent with the hypothesis that future movements arise as the result of two interacting processes: a conventional trial-by-trial error-correction mechanism, overlaid with a process that exhibits long memory. The existence of this long-memory process suggests that future efforts to describe motor learning should include models more complex than simple state-space (ARMA) formulations.

## Results

The control of predictive-saccade amplitudes was examined by asking subjects to perform simple saccade tasks that involved looking back and forth between two targets at a fixed rate while their eye movements were recorded (see Methods). Within each block of trials, primary-saccade amplitudes were measured and compiled sequentially to form a time series. Corrective saccades were not considered here, since they may be driven by different motor-learning mechanisms and may therefore constitute a separate learning process [1], . Primary-saccade amplitudes were analyzed for evidence of two major processes. First, using a short predictive-saccade task involving only 300 trials in a block (Task 1), we looked for the existence of a simple trial-by-trial process that compensates for errors on each trial by immediately updating the response on the next trial. It has previously been suggested that the motor system can adapt according to the error experienced in the single preceding trial, and does not require a consistent error signal across many trials [23]. It seemed likely that a similar process could modulate learning for predictive saccades. Second, using predictive-saccade sessions from a longer task that involved either 500 or 1000 successive trials (Task 2), we looked for evidence of a long-term process that modulates movements by monitoring performance during the several previous trials. Such processes are important because systems that learn solely on a trial-by-trial basis have the potential to become oscillatory or unstable. Aggregating performance information across many past trials provides a way to compensate for systematic trends of the stimulus perturbation with less concern for inter-trial variability – for example, this process might be engaged more heavily when responding to a constant displacement of the stimulus as would occur in typical adaptation paradigms. By not restricting the duration across which inter-trial correlations were examined, we simultaneously searched for evidence of either simple state-space (ARMA) processes or more complex long-term dynamics.

### Evidence for a trial-by-trial error correction process

Subjects were asked to perform three short blocks of only 300 trials each (Task 1) in which, throughout the block, a stimulus perturbation was presented on either 0%, 10%, or 20% of the total number of trials (Figure 1A). This provided a means to introduce stimulus variability (in the form of catch trials) while encouraging subjects to generate predictive saccades, as variability in the form of stimulus noise added to the target position on every trial hindered this task [24]. Note that since subjects were generating predictive saccades, they observed a catch trial as a post-saccadic error that was larger than expected; thus, any effect of catch trials had to occur in the correction made on the following saccade. Catch trials did not disrupt prediction: for each block, saccadic latencies lay in the predictive range (0%: −2.02±69.80 msec, 10%: −6.65±77.69 msec, 20%: −13.69±76.99 msec). Across all subjects, the latencies of saccades on catch trials did not differ from the latencies of saccades on all other trials (t-test, p>0.10 for all subjects), and, with the exception of one subject in the 10% catch-trial condition, the latencies of the saccades immediately following the catch trials did not differ from those of the other saccades (t-test, p>0.16 for all other subjects and catch-trial conditions). Furthermore, there is no difference between latencies in the 0% and 10% catch-trial conditions or between the 10% and 20% catch-trial conditions; saccade latencies are only significantly different between the 0% and 20% catch-trial condition although the change in latencies is small (only 11 msec; Kruskal-Wallis one-way ANOVA based on ranks with pairwise comparisons using Dunn's method, p<0.05). Thus, catch trials were a reasonable method of exaggerating spatial performance errors without disrupting the predictive process. Since catch trials did not occur on consecutive trials, these perturbations were used to examine trial-by-trial learning.

**Figure 2:**
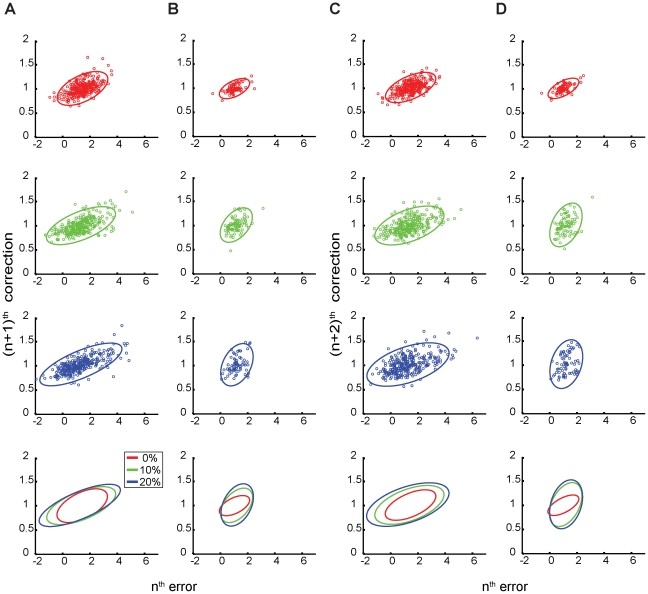
Trial-by-trial error corrections in the stimulus variability task. A: Predictive-saccade data from one representative subject during Task 1, reflecting corrections to errors made on the very next primary saccade (the (n+1)^th^ saccade, or, in the case of catch trials, the return saccade from the displaced target). B: Reactive-saccade data from one subject in the control for Task 1, for the (n+1)^th^ correction. In C and D, the same time series are used to plot the corresponding predictive-saccade and reactive-saccade error corrections made on the (n+2)^th^ saccade, or the next saccade made in the same direction as the current saccade. In all cases, the top three panels show data from the three stimulus-variability conditions (from top to bottom, the 0% catch-trial, 10% catch-trial, and 20% catch-trial conditions); the bottom panel shows the superimposed CE_95_ ellipses from the top three plots for comparison purposes.

Trial-by-trial corrections were analyzed by plotting the error made on the current trial (the n^th^ saccade) versus the gain change on the next primary saccade (amplitude of the next saccade divided by the amplitude of the current saccade), then fitting a 95% confidence ellipse (CE_95_) to the data. The parameters of the CE_95_, particularly the angles of the major and minor axes, describe trends in the data. Specifically, the CE_95_ major-axis angle (slope of major axis) reflects how well the system compensated for errors by conveying the quality of the gain correction produced as a function of the error size. Examples of data fitted with CE_95_ ellipses for both predictive saccades and reactive saccades are exhibited in Figure 2.

Depending on how “next trial” is defined, there are two trial-by-trial corrections of interest that compensate for errors on the n^th^ trial. First, there is the correction on the return primary saccade, or the saccade made away from the target where the error occurred (that is, the (n+1)^th^ saccade). By comparing the angles of the CE_95_ major axes in each of the stimulus-variability conditions, we assessed modulation of the error-correction process as artificial errors were introduced. In this case, all errors – including errors induced by catch trials – were well compensated on the (n+1)^th^ saccade; there were no significant differences between the CE_95_ axis angles for all variability conditions (paired t-test between the 0% and 10% catch-trial condition, p = 0.88; paired t-test between the 0% and 20% catch-trial condition, p = 0.54; see Figure 2A and Table 1). In other words, whether subjects were observing their own prediction errors or the artificially induced errors resulting from the catch trials, they corrected all errors in the same manner. Otherwise, there would have been a change in the CE_95_ angle as greater frequencies of catch trials caused changes in the error-correction process.

**Table 1:** CE_95_ major axis angles for all catch trial (CT) conditions.

	(n+1)^th^ correction			(n+2)^th^ correction		
Subject	0% CT	10% CT	20% CT	0% CT	10% CT	20% CT
A	−3.02	−3.00	−3.00	−3.02	−2.98	−3.03
B	−3.04	−3.05	−3.03	−3.05	−3.06	−3.04
C	−3.04	−3.06	−3.07	−3.03	−3.06	−3.09
D	−3.02	−3.03	−3.02	−3.08	−3.07	−3.07
G	−3.04	−3.04	−3.03	−3.06	−3.06	−3.06
J	−3.04	−2.99	−2.99	−3.04	−2.97	−2.99
React	−3.05*	−3.06*	−3.07*	−3.01*	−3.05	−3.07

For the predictive-saccade data, the 10% catch trial and 20% catch trial conditions were not significantly different from the 0% catch trial condition, paired t-test, p>0.45.

*Significant difference between predictive and reactive CE95 with p<0.05.

As further evidence of this, we compared these findings against data from two subjects who participated in a separate control experiment involving the generation of reactive saccades. In the 0% catch trial condition (that is, without stimulus perturbations), there was a significant difference between the CE_95_ angles for predictive and reactive tracking (t-test, p = 0.02). Furthermore, when catch trials were introduced, the reactive-saccade CE_95_ angles changed greatly compared to the reactive-saccade 0% catch trial condition (Figure 2B) because each reactive saccade was simply made in response to the visual target and was therefore independent of previously generated reactive saccades. This resulted in a change in how catch trials affected both the size of errors and the types of corrections made. Indeed, the reactive-saccade CE_95_ angles remained significantly different from those of predictive saccades (10% catch trials, t-test, p = 0.03; 20% catch trials, t-test, p = 0.01; Table 1).

Unfortunately, there was a potential confound when examining corrections made on the (n+1)^th^ trial in response to catch trials. Since the catch trial was in the form of a displaced saccadic target, the return saccade must necessarily have been larger to bring the eyes back to the next non-displaced target. Thus, this “compensation” might not reflect learning at all, but simply the appropriate response to a larger requested saccade. To clearly demonstrate that trial-by-trial learning does occur, therefore, it was necessary to consider an alternative definition of the “next” trial. For that, we turned to the motor learning literature.

With respect to adaptation, subjects are capable of changing the gains of rightward and leftward saccadic movements in different ways: subjects can adapt in a gain-increase manner in one direction and a gain-decrease manner in the opposite direction, or only adapt saccades moving in one direction while maintaining a fixed gain in the other direction [25]–[29]. It is possible that error corrections occur in a direction-specific manner for predictive saccades as well; that is, subjects might have learned that a catch trial (which produced a large, unexpected error) occurred when making a rightward saccade, so they would then anticipate the need to make a much larger saccade the next time they looked to the right (see Figure 1A, lower panel). Therefore, we considered as an alternative definition of “next trial” the (n+2)^th^ saccade, or the next saccade made in the same direction as the current saccade. This eliminated the problem inherent with catch trials since these corrections took place during saccades that were made between two non-displaced targets, so the only reason for compensation was learning from the previous saccade. Such a corrective mechanism was also of interest because it would indicate that direction-specific learning may be a common feature of motor learning.

Akin to the data observed for the (n+1)^th^ correction, error-correction performance on the (n+2)^th^ saccade was consistent across all catch-trial conditions as demonstrated by the presence of similar CE_95_ axis angles (paired t-tests between the 0% and 10% catch-trial condition, p = 0.65; 0% and 20% catch-trial condition, p = 0.45; see Figure 2C and Table 1). As before, these CE_95_ axis angles were also significantly different from those of reactive saccades in the 0% catch trial condition (Figure 2D; t-test, p = 0.01). This implies that subjects actively corrected performance errors – including artificially induced errors due to catch trials.

**Figure 3:**
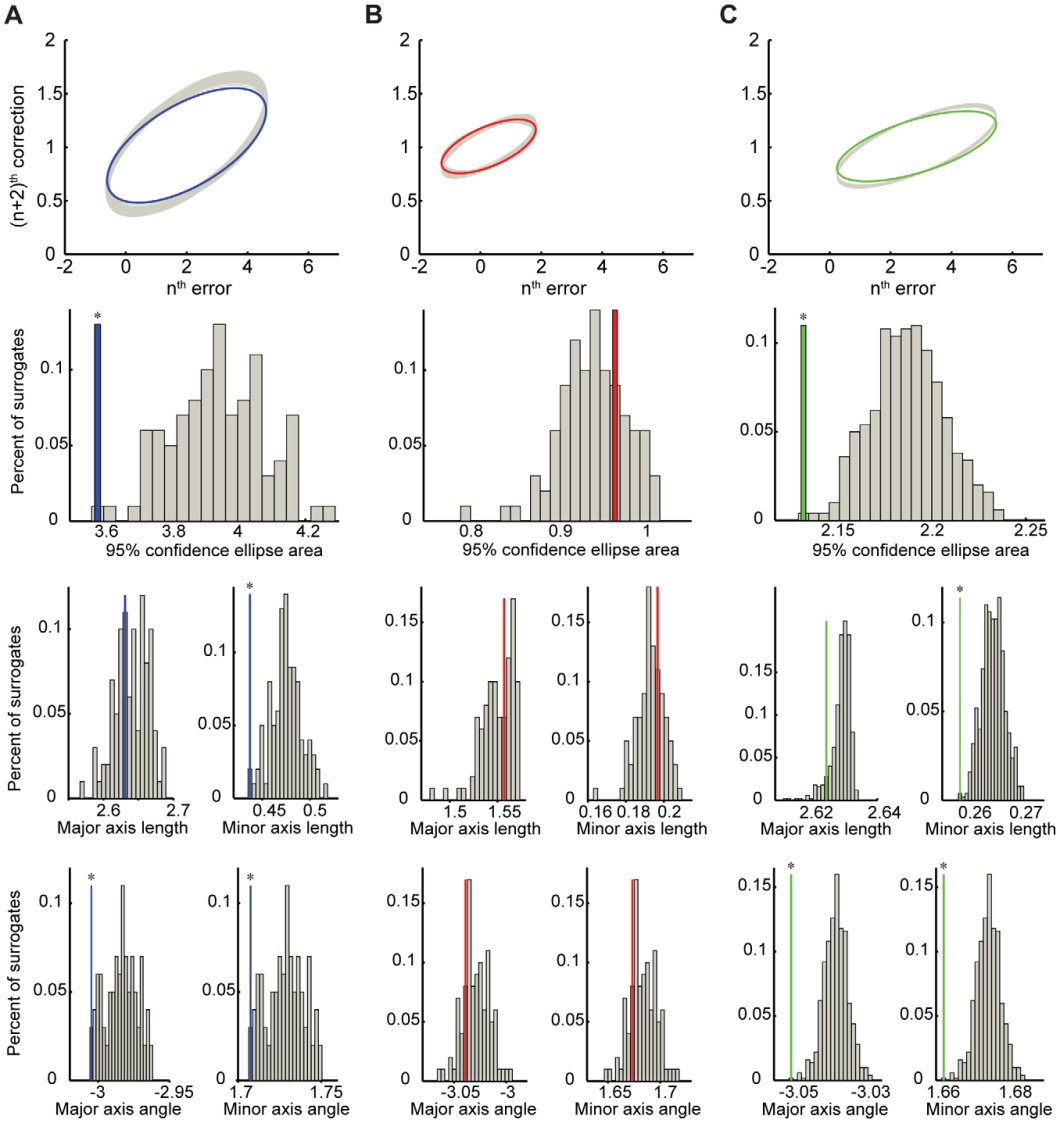
Surrogate data analysis of trial-by-trial error corrections. Data show one representative time series from predictive-saccade tracking during Task 1 (A, blue), reactive-saccade tracking in the corresponding control task (B, red), and simulation data from an ARFIMA(0,*d*,0) process (C, green). In all cases, the top panel shows the CE_95_ for the actual data (thick colored line) and the generated set of surrogates (multiple overlapping gray ellipses). The lower three panels show comparisons between the data and surrogates across the five characteristic CE_95_ parameters (from top to bottom): the ellipse area, major and minor axis lengths, and major and minor axis angles. The gray bars on the histogram plots are the data from the surrogate time series; the single colored bar on each histogram is the actual time series data, which has been rescaled vertically for clarity. Significant differences at greater than the p = 0.05 level are indicated by (*).

**Figure 4:**
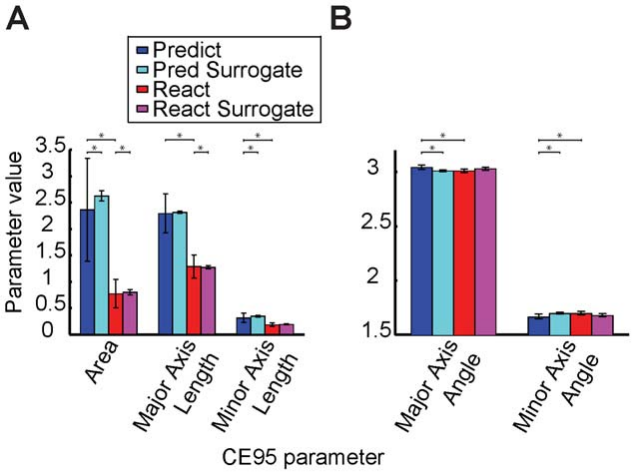
Group data summary of the surrogate data analysis. For all subjects, the five average CE_95_ parameters are displayed (A, B) along with error bars (S.D.). We present the absolute value of the CE_95_ major axis angles (first set of bars in B) for graphing purposes; these angles are reported as negative in the text. Significance between pairs of parameters at greater than the p = 0.05 level is indicated by (*). The predictive-saccade data (blue) are, in general, significantly different from the surrogate data (cyan) and the reactive-saccade data (red) across many of the CE_95_ parameters. Reactive saccades, however, are not very different from their surrogates (magenta).

Despite the differences between predictive and reactive saccades, it might be argued that trial-by-trial error corrections could have arisen by chance. That is, if predictive-saccade amplitudes were randomly chosen as independent trials, a saccade with a particularly large or small error would tend to be followed by a saccade with a more moderately-sized error just by chance; this could have resulted in a process that only appeared to be governed by an active error-correction mechanism. To investigate this, we analyzed surrogate data sets. Each surrogate time series was produced by randomly shuffling the order of predictive-saccade amplitudes in the original data, which removed all temporal correlations without changing the distribution of saccade amplitudes in the series. The error-correction analysis was then performed on these data sets (that is, current errors and next-trial error corrections were assessed for each surrogate). Surrogate CE_95_ values were observed to lie along the ideal compensation angle, which would arise either by assuming that trials were independently drawn from a Gaussian distribution or that there was complete compensation for errors on every trial (see Methods). In this case, the ideal angle of the CE_95_ major axis was approximately -3.01 radians. This analysis was restricted to only the 0% catch trial condition for the (n+2)^th^ correction. Each subject's data were compared against 1000 surrogate time series. CE_95_ values were examined in terms of the five characteristics that fully describe an ellipse (single-subject data in Figure 3; group data summarized in Figure 4): the ellipse area, the lengths of the major and minor axes (which roughly describe the range of saccade errors made across trials and the scatter about the trend in the data, respectively), and the orientations (angles) of the major and minor axes (which reflect the quality of the compensation mechanism). Since ellipse axes must be orthogonal, we only reported findings for the CE_95_ major axis angles below.

As expected, all the surrogate data were found to yield CE_95_ values whose orientations were not significantly different from the ideal compensation angle (average surrogate CE_95_ angle was -3.01±0.01; t-test, p = 0.71). Furthermore, reactive-saccade data, with an average CE_95_ angle of -3.01±0.02, were not significantly different from either the corresponding surrogate data (t-test, p = 0.35; Figure 3B) or the ideal angle (t-test, p = 0.83). Predictive saccade data, on the other hand, bore little resemblance to either one. The average CE_95_ angle was -3.04±0.02, which was significantly different from the ideal angle (t-test, p = 0.01; Figure 4B). On an individual basis, the CE_95_ values for each subject's predictive saccades were found to be significantly different from those for their respective surrogate data (paired t-test, p<0.01; data from one subject is displayed in Figure 3A). Interestingly, the predictive-saccade ellipses were also significantly narrower than were the surrogates (that is, the ellipse minor-axis lengths were shorter) (paired t-test, p = 0.01; Figure 4A). As the CE_95_ minor axis describes the scatter or variability about the main trend in the data, finding shorter minor axes for the predictive-saccade time series implies that the error-correction was actively controlled, not random. In contrast, major-axis lengths were not significantly different (paired t-test, p = 0.29), reflecting the fact that the range of errors was the same in both the actual and the surrogate data.

Although errors were actively corrected, it was somewhat surprising to observe that these corrections were less than complete (that is, the CE_95_ axes were tilted less steeply than the ideal tilt angle, or directed more toward the abscissa, which implied that errors were not fully corrected to the mean saccade amplitude on each trial). In fact, such a situation actually is preferred: compensating solely and completely for errors experienced in the single previous trial could lead to oscillations and potential instability. While the controlling process might have achieved this incomplete compensation by decreasing the amount of learning that took place in response to each individual error (for example, by decreasing the learning rate in a single-state, state-space (ARMA) model), an alternative approach to yield this same under-correction could be to utilize performance information from more than one previous trial. To examine this possibility, it was necessary to search for evidence of a process operating across a longer timescale.

### Evidence for a long-term process

Inter-trial correlations extending beyond one trial were explored with simple time-series analysis techniques – in particular the power spectra, which describe how similarities between trials vary at different time scales. The power spectrum – along with its Fourier-transform pair, the autocorrelation function [18], [30] – conveys how information is retained between saccades separated by one or more trials. Using these analyses, it was possible to assess the number of past trials over which the system utilized performance information. Proper application of such techniques requires lengthy, continuous sequences of saccades; therefore, we had subjects in Task 2 make predictive saccades to either 500 or 1000 targets, and explored the inter-trial correlations in these longer data sets.

**Figure 5:**
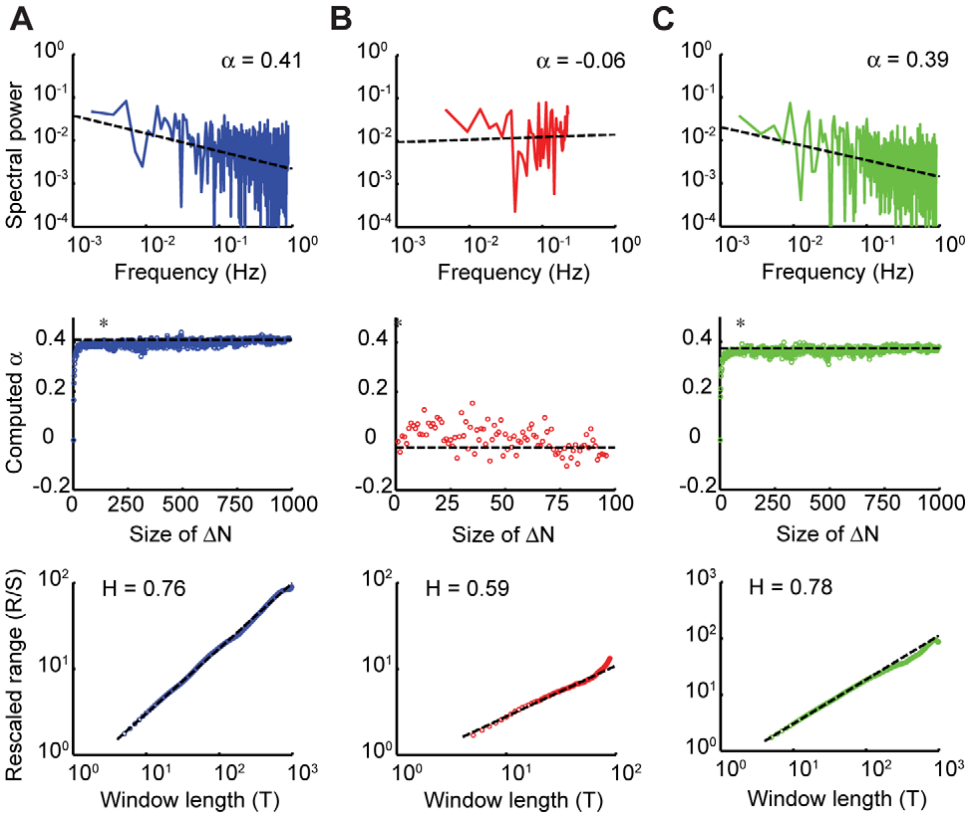
Analysis of long-memory processes. Predictive-saccade data from one representative subject in Task 2 (A) is compared against a short sequence of reactive-saccade data (B). The same analyses were also applied to simulation data from an ARFIMA(0,*d*,0) process (C). In all cases, the top panel is the power spectrum of the data plotted on a log-log scale, with the measured α value reported. The middle panel is the bootstrap analysis, demonstrating the change in the value of α from random (near zero) to its measured value (reported in the top panel) as the shuffling-block size, ΔN, is varied. The first point at which the value of α is no longer significantly different from the value measured in the top panel is indicated by (*). The bottom panel displays the result of the Hurst rescaled-range analysis, the slope of which is used to estimate the *H* parameter. Whereas the predictive-saccade data and the simulation data exhibit long memory, the reactive-saccade data do not.

The power spectrum for predictive saccades appeared roughly linear on a log-log plot (data from one subject, Figure 5A); that is, for frequency *f*, 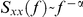 (power-law decay). The exponent α is the frequency scaling exponent. This power-law decay of the power spectrum is often associated with power-law decay of inter-trial correlations as assessed by the autocorrelation function [31]. Since inter-trial correlations decayed in this manner, this meant that more information was retained from one trial to the next than there would be in the case of a typical state-space (ARMA) process, for which inter-trial correlations decay exponentially. In addition, power-law decay indicated that control of the current saccade was modulated by performance errors far in the past (fluctuations were observed across all time scales). This suggested that the control of predictive-saccade amplitudes might be governed by a long-memory process [32].

From the power spectra, the frequency scaling exponent, α, was measured for each time series (see Methods). This α value can be used to characterize the nature of the long-range dependence present in the data. The average measured α value for all subjects was 0.37±0.14, which was significantly different from either zero or one (t-test, p<0.01). A slope of zero of the power spectrum on a log-log plot is suggestive of a random white-noise process, in which there are no inter-trial correlations. Reactive saccades exhibited this feature (Figure 5B; average α = -0.05±0.04), which makes sense given the trial-by-trial analysis also suggested that reactive saccades resemble a random process. Values of α greater than zero but less than one imply that the process is persistent, in which large values tend to follow large values and small values follow small values. Such persistence is consistent with the trial-by-trial analysis that suggested that errors on each trial were under-compensated. In such a circumstance, it would take several trials to fully correct for a given error, meaning that groups of successive saccades would tend to all be consistently larger or smaller than the mean saccade amplitude. In contrast, if α was less than zero the process would be considered anti-persistent, in which large values tend to follow small values and vice versa.

**Figure 6:**
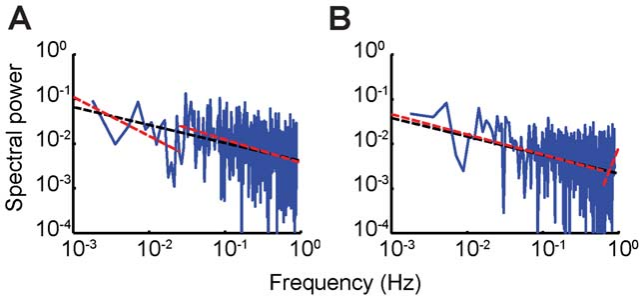
Piecewise versus single linear regression analysis of the power spectrum. A, B: Two representative power spectra showing the comparison between the piecewise-linear fit and the single-linear fit. In both cases, the single-linear fit is found to better fit the data according to the Bayesian Information Criterion, suggesting that the power spectra exhibit power-law decay.

Although the power spectra appeared linear on a log-log plot, these spectra were quite noisy. Thus, we tested the hypothesis that the data could instead have been generated by a state-space (ARMA) process by asking if the power spectrum contained an inflection point. An inflection point might be expected if inter-trial correlations decayed exponentially such as might be observed for a state-space (ARMA) process that forms a low-pass filter, which is characterized by a flat power spectrum in the low-frequency range [33]. We approximated this using a piecewise-linear regression and allowed the model to pick the best inflection point as well as the slopes of the piecewise regressions (sample piecewise fits are exhibited in Figure 6; see Methods). Such an approach is conservative since it is also possible that a long-memory process with two different scaling regions would be misclassified as a state-space (ARMA) process according to this method, particularly since the slope of the low-frequency region was not restricted to zero. Nevertheless, in all cases a single regression line was found to fit the data better according to the Bayesian Information Criterion (see Methods; difference in the Bayesian Information Criterion fitting values was strongly in favor of a single linear regression over a piecewise linear regression; t-test, p<0.01). This indicated power-law decay of the power spectra.

One question that immediately arose upon finding evidence of a long-memory process was exactly how far into the past trials were correlated. Power-law scaling suggests that trials were related infinitely far into the past; however, this seemed biologically implausible because it implied that the brain was keeping track of performance errors across thousands of previous saccades. To assess the extent of the long-range dependence, we used a modification of the bootstrap technique developed by Wang et al. in which the data were divided into blocks of size ΔN and subsequently shuffled [34]. This eliminated correlations between trials on timescales greater than ΔN. For very small values of ΔN, the computed value of α tended toward zero since shuffling reduced the time series to a random process. Thus, by finding the smallest value of ΔN for which α was not different from that value measured for the original time series, it was possible to estimate the extent of inter-trial correlations (Figure 5). For our predictive-saccade data, this value of ΔN was, on average, 73 trials (significantly different from zero, t-test, p = 0.01); in contrast, for reactive saccades the average ΔN was 7.7 (not significantly different from zero, t-test, p = 0.28). Therefore, for these data, long memory truly implied a long time – on the order of 100 saccades – across which the motor system stored performance errors and used that information to drive future predictive movements.

Evidence of long memory was confirmed by two other independent analyses. The first involved computing the Hurst exponent (*H*), also known as the temporal scaling exponent. Mathematically, the temporal (*H*) and frequency (α) scaling exponents obey the relationship *H* = (1+α)/2, although both were computed by completely different techniques (see Methods). Thus, the computation of both *H* and α provided a means to verify that predictive-saccade amplitudes were indeed controlled by a long-memory process. For predictive saccades, the average *H* value was 0.73±0.07 (representative data from one subject are shown in Figure 5A). This again was indicative of a persistent process, since 0.5<*H*<1 (*H* was significantly greater than 0.5; t-test, p<0.01). Furthermore, measured *H* values from the predictive-saccade data did not significantly differ from those computed using the measured α values, which verified the findings of each method (t-test, p = 0.07). Reactive-saccade data (Figure 5B), on the other hand, were likely to reflect a random process, as the measured *H* value of 0.57±0.18 was not significantly different from 0.5 (t-test, p = 0.53).

The second analysis technique used to verify the finding of long memory was to compare a short-memory and a long-memory model. The two model classes were ARMA (state-space) models and the related long-memory ARFIMA models (see Methods and Methods S1). In comparing fits across all non-catch trial data sets collected in both tasks, it was found that an ARFIMA model fit the data best in 11 out of 16 cases according to the Bayesian Information Criterion (Table 2). For those 11 cases, the average computed *d* value – the ARFIMA model parameter that described the long-memory process – was 0.22±0.06. The value of *d* is related to the Hurst exponent, *H*, by the relationship *d* = *H* – 0.5; thus, this value of *d* was consistent with the measured *H* and α values found above (measured and computed *d* values were not significantly different; t-test, p = 0.58). Furthermore, this measured value of *d* was significantly different from zero, which indicated that these data did not come from either a random process or an ARMA (state-space) process (t-test, p<0.01). Thus, the model-fitting analysis also confirmed the piecewise-linear power-spectrum fits. Together, these analyses strongly supported the notion that motor learning exhibits long memory.

**Table 2:** ARMA and ARFIMA model fits.

Subject	Paradigm	Best Model Fit (BIC)	Estimated *d* value
A	Task 1	ARFIMA(0,*d*,0)	0.2
B	Task 1	ARFIMA(0,*d*,0)	0.24
	Task 2 (1000)	ARMA(2,0)	−
C	Task 1	ARFIMA(0,*d*,0)	0.24
	Task 2 (500)	ARFIMA(0,*d*,0)	0.23
D	Task 1	ARFIMA(1,*d*,1)	0.32
	Task 2 (500)	ARMA(2,3)	−
	Task 2 (1000)	ARFIMA(2,*d*,1)	0.24
E	Task 2 (1000)	ARMA(2,1)	−
F	Task 2 (1000)	ARFIMA(1,*d*,1)	0.24
G	Task 1	ARFIMA(0,*d*,0)	0.25
	Task 2 (500)	ARFIMA(0,*d*,0)	0.13
	Task 2 (1000)	ARFIMA(0,*d*,0)	0.21
H	Task 2 (500)	ARMA(1,0)	−
I	Task 2 (500)	ARFIMA(1,*d*,1)	0.08
J	Task 1	ARMA(0,0)	−
Average			0.22±0.06

### Comparison of data to a simulation of a long-memory process

We also used the ARFIMA model to produce simulated time series. Since we were interested in investigating primarily the long-memory process in isolation, we chose to simulate an ARFIMA(0,*d*,0) process – that is, a process that did not contain any additional short-memory ARMA (state-space) processes. Using the measured *d* value of 0.22 from the data, we produced 100 simulated data sets; every time series contained 1000 trials. For each simulated time series, we measured the temporal and frequency scaling exponents (data for one simulation is displayed in Figure 5C). On average, the simulations yielded α = 0.40±0.06 and *H* = 0.75±0.02, which corresponded well to the values in the predictive-saccade data: simulated and measured exponents were not significantly different (α: t-test, p = 0.19; *H*: t-test, p = 0.27).

Conducting the bootstrap analysis above, we estimated the length of time over which inter-trial correlations were present by measuring ΔN for each simulated series. The simulation data yielded an average ΔN value of 93 trials, which is not significantly different from the measured ΔN values for the predictive-saccade time series (t-test, p = 0.47). This bootstrap analysis confirmed that inter-trial correlations extended to somewhere on the order of 100 trials into the past; in other words, an error made 100 saccades ago (about 40-50 seconds in the past) still affected the planning of the current predictive saccade.

Finally, we examined trial-by-trial corrections in these simulation data by looking at how errors were corrected on the (n+2)^th^ trial. For each time series, we compared the CE_95_ measured for the simulated data against a corresponding set of surrogate data; the analysis from one sample simulated time series was presented in Figure 3C. In all cases, the simulated data behaved similarly to the predictive-saccade data. We found that errors appeared to be well corrected in the simulations. There was a significant difference between the CE_95_ axis angles and those of the corresponding surrogate data (for all simulated time series compared to their respective surrogates, p<0.01), which manifested as a less steeply oriented CE_95_; this again suggested that errors were under-corrected. Such a finding was reasonable because the simulated data were all persistent time series, so that trials were more similar to recent trials in the past than chance (less “corrected”). In fact, this under-correction was so robust in the ARFIMA model data that we found the simulated data CE_95_ axes to be oriented even less steeply (i.e., errors were more under-corrected) than for the actual predictive data (t-test, p<0.01). Since these simulations did not explicitly contain any short-memory processes (that is, the ARMA coefficients of the model were zero), the simulated data exhibited only persistent behavior and little trial-by-trial corrections. Therefore, these findings indicated that there was likely to be an active trial-by-trial error-correction mechanism working alongside the long-memory process in the control of predictive saccades. Together, these counter-balancing processes produce behavior that maintained a reasonable level of accuracy by learning from errors on a trial-by-trial basis, without the loss of performance stability that comes from failing to examine average performance across many trials in the past. Thus, we consider the control of predictive saccades to arise from some combination of performance information between a short-term mechanism that corrects errors and a long-term, persistent process that provides performance stability.

## Discussion

The control of predictive-saccade amplitudes is regulated as a motor learning process, in that prior performance errors modulate future behavior. This process, unlike a typical adaptation paradigm, has the advantage of being statistically stationary, such that the movement gain (and to a rough approximation, the variability of that gain) does not change throughout a session. Such features allow for the application of standard analysis techniques to quantify inter-trial correlations and identify underlying dynamics. We hypothesize that the predictive-saccade task utilizes an error signal derived as the difference between predicted and observed movement outcomes. Such an error signal has previously been demonstrated to drive motor adaptation [1], suggesting that this predictive error signal may be common to the phenomenon of motor learning. Therefore, it may be possible to extrapolate the findings regarding this simple predictive-saccade task to other, more complex motor learning processes that are nonstationary, such as motor adaptation in response to an artificially-induced stimulus perturbation.

Note that in conducting our analyses, we assume that movement error is the critical quantity being retained to inform future movements. Evidence from the trial-by-trial analysis supports this idea, since subjects appear to respond principally to movement errors; such errors are actively and appropriately corrected (partially) on the next trial. As noted above, there is also some evidence from the motor adaptation literature that the information utilized to drive learning is composed of motor errors [1], [35]-[36]. Indeed, many current modeling efforts assume that movement error – often represented as the difference between ideal and actual movement outcomes – drives learning. Thus, although the exact nature of the information being retained by the motor system to plan and generate predictive saccades is yet to be investigated, it seems reasonable to assume that the motor system retains at least some performance error information when generating future movements.

This error information appears to drive predictive saccades through two coexisting processes: a trial-by-trial error-correction mechanism that acts to maintain accuracy, and a persistent long-memory process that reduces fluctuations by aggregating performance errors across many previous trials. While we found clear evidence suggesting that the trial-by-trial error correction process is direction-specific, we cannot rule out the possibility of a second trial-by-trial mechanism that is not direction specific. Nonetheless, the presence of a direction-specific learning process is intriguing since motor adaptation is also known to take place on a direction-specific basis [25]-[27], [29].

In contrast to the predictive-saccade findings, a simple reactive-saccade task exhibits quite different features. Since each reactive saccade is generated independently in response to a novel visual target, it is not necessary to retain information from one trial to the next. The result is a random, white-noise process, as indicated both by the lack of difference between the data and surrogates in the trial-by-trial CE_95_ analyses as well as by the measured temporal and frequency scaling exponents in the long-term correlation analyses. The errors made during a predictive-saccade task, on the other hand, are directly relevant to the production of the next predictive saccade because each saccade is made to an estimated target position in anticipation of the stimulus appearance. Thus, it is necessary to learn from prior performance errors, resulting in significant differences between the inter-trial correlations of reactive and predictive saccade data as assessed by several distinct analysis techniques. By utilizing information about past performance, the motor system is able to produce consistent, automatic, anticipatory behavior that takes little effort or conscious input.

Evidence for the presence of a long-memory process is quite striking; three independent analyses confirm this finding. This has significant implications for motor learning in general. In particular, it calls into question some current efforts to model the motor learning process. Such models, applied to explain gross changes in behavior such as the adaptation of a movement gain state in response to an artificially induced stimulus perturbation, expend much effort to fit the nonstationary trend and ignore information about underlying dynamics that may reside in the “variability” about the trend. This results in state-space (ARMA) models that require time-dependent parameters [17] – a problem inherent to fitting a nonstationary process with a stationary model. Our results – based on the analysis of a relatively stationary process – confirm that it is inappropriate to model at least one aspect of motor learning with a state-space (ARMA) model. We find power-law decay of inter-trial correlations, which cannot be captured in the exponential decay of information between trials inherent in a state-space (ARMA) model. Additionally, state-space (ARMA) models require explicitly defining the number of time scales on which learning takes place, whereas long-memory processes imply learning on all time scales, as demonstrated by the presence of significant fluctuations at all frequencies of the power spectrum (power-law decay of the power spectrum). These findings suggest that we must turn to alternative models to describe the complexities of motor learning – models that must be carefully applied to nonstationary phenomena such as motor-adaptation tasks.

Unfortunately, the existence of long memory is objectionable for exactly that property that characterizes it – the existence of many time scales. This implies that the brain must maintain information about performance errors over extremely long intervals of time; the bootstrap analysis we performed suggests that the brain might use information from as many as 100 prior trials when planning the next movement. Are such processes feasible for the brain to implement? In fact, power-law processes have been demonstrated at numerous levels throughout the nervous system. At the neural level, these processes result from ion channel kinetics, whose effects may be strong enough to potentially explain measureable behavioral responses such as the tilt aftereffect, a prominent visual illusion [37]-[39]. Long memory has been described for many other biological phenomena as well; for example, it has been suggested that the finding of long memory is evidence of a healthy heart rate, and that a loss of such complex dynamics is a sign of an impending arrhythmia or heart failure [40]-[41]. Therefore, it is certainly feasible that the control of motor learning is influenced by a process with long memory.

The features of long memory can be produced or mimicked by the existence of multiple, interacting short-memory (state-space, ARMA) processes. For example, if one process drives changes in the parameters that govern the second process, it is possible to produce a set of data that appear to exhibit long-range dependence [42]-[45]. Thus, while a single state-space (ARMA) model is not sufficient to describe motor learning dynamics, it may be possible to do so using coupled ARMA processes. Such a model design is also in line with the notion of two dominant time courses of learning [14], [46]-[49]; two ARMA processes, operating at different timescales, might be sufficient to mimic this behavior while producing fluctuations that appear to exhibit long memory. Such a solution may be more biologically plausible than a single long-memory process.

Interacting state-space (ARMA) processes could occur as a result of converging information from multiple brain regions, each performing separate calculations at different temporal rates. Some portions of this widespread motor learning network have already been identified. Certainly, the cerebellum plays a major role in both adaptation and prediction; patients with cerebellar deficits exhibit difficulties in saccade adaptation and prediction tasks [50]-[54]. The cerebellum is thought to contain a forward model which generates predictions [55]-[56], which could serve a crucial function in all motor learning processes. However, the involvement of other brain regions such as the frontal and parietal lobes in prediction tasks [57], along with the superior colliculus [58] and especially the cerebellar thalamus [59] in adaptation tasks, suggests the presence of a dispersed network. Working together, these regions may monitor numerous factors influencing the control of movements such as changes in the environment and changes within the body, helping the motor system determine how best to address movement errors. Integration of the outputs from these multiple brain regions could be sufficient to produce behavioral data that appear to exhibit long memory. A more thorough investigation is required to explore the feasibility of this proposal. Nevertheless, it is important to recognize and actively examine data for the presence of such complex dynamics as long memory, which cannot be represented using the simple state-space (ARMA) models that are currently employed to describe motor learning. In doing so, we may find that the influence of such a long-memory process is the net result of trying to balance the need for immediate compensation for errors with the stability necessary to maintain accurate long-term performance. In that sense, it would not be surprising to find hints of long memory in all sensorimotor tasks.

## Materials and Methods

### Participants and ethics statement

Eye movements were recorded from ten subjects who participated in one or more of three experimental tasks. Written informed consent was obtained from each participant. Experiment protocols were approved by the Western Institutional Review Board under contract with the Johns Hopkins Medical Institutions, where all studies were conducted. Subjects A-D and G performed the stimulus variability task (Task 1). Subjects D and J participated in a control version of this task. Subjects B-I performed either one or both versions of the extended tracking task (Task 2). Subjects who participated in more than one task experienced each task at least a month apart. Only subjects D and E were not naïve to the purposes of this study.

### General Methods

Data were acquired on a PC-compatible Pentium 166-MHz computer running real-time experiment control software developed in-house. Eye movements were recorded using a directional scleral search coil (Skalar Medical BV, Delft, The Netherlands) to record horizontal and vertical eye movements at 1000 Hz from either the right or left eye [60]. Scleral coil data were digitized with a 12-bit analog-to-digital converter, setting the system resolution to about 0.03°. Subjects sat in a dark room in a stationary chair, and a bite-bar was used to minimize head movements. Targets were displayed using two methods. In Task 1 and the short version of Task 2, targets were generated by rear-projecting a mirror-controlled laser dot onto a screen 1 m in front of subjects, producing a target that was 2 mm in diameter. For subjects performing the long version of Task 2, targets were LEDs placed at fixed locations prior to the start of the experiment.

Analyses of eye-tracking data were done off-line with an interactive computer program that selected primary saccade start- and end-points using a velocity threshold of 15°/sec (typical threshold values in the literature range from 10°/sec to 40°/sec; see also [61]), which were then visually confirmed prior to analysis. Sample trajectories of saccades demarcated using this threshold are illustrated in Figure 1A. When subjects blinked during a saccade, the resulting saccade was discarded from analysis. Primary saccade amplitudes were measured, then arranged to form a time series.

### Tracking Tasks

Task 1 was a short tracking task to examine trial-by-trial error corrections. Subjects performed three blocks of trials, each consisting of 300 target presentations paced at 0.9 Hz (inter-target interval: 556 msec). Each block contained a different level of target variability, which was introduced to exaggerate spatial trial-to-trial errors. Since variability in the form of spatial noise added to every trial decreases the ability to make predictive saccades [24], controlled variability was introduced in the form of pseudo-randomly interspersed catch trials in the otherwise predictable task (Figure 1A, lower trace). In normal trials, targets consistently appeared ±5° on either side of the vertical midline; during a catch trial, the target was displaced 2° farther from the midline. Catch trials were restricted such that they could not occur on successive trials; this enabled the examination of the effect of a single catch trial on the next trial. By changing the proportion of catch trials in each block, target variability was modulated; subjects experienced one of each block containing 0%, 10%, or 20% catch trials. Subjects experienced blocks in order of increasing variability to reduce the chance that expectations of experiencing catch trials would influence error corrections in future blocks; subjects were not informed of the catch-trial perturbations. The control task for this paradigm repeated the same stimulus-variability conditions but for 100 trials paced with longer, random inter-stimulus intervals (mean 1500 msec) to promote reactive tracking. The 0% catch trial condition was also used as a control to obtain a set of reactive saccades to compare against Task 2.

Task 2 was an extended tracking task, in the sense that subjects were asked to generate predictive saccades for greater numbers of successive trials. Subjects made saccades to alternating targets appearing at ±5° on either side of the vertical midline, paced at 0.9 Hz for 500 trials. We also ran an even longer version of this task in which subjects were asked to track alternating targets paced at 1 Hz for 1000 trials (the slightly increased frequency was used to keep the total duration of the block reasonable even though the number of trials doubled; targets during these trials appeared ±10° on either side of the vertical midline). This longer version enabled us to verify the findings from the shorter versions; that is, to ensure that there was no high-end cutoff in the extent of inter-trial correlations beyond 500 trials. In either version, target timing and position were completely predictable.

In all tasks, subjects were given no explicit instructions as to the timing or accuracy of their movements; they were simply asked to “look at the targets.”

### Analysis Techniques

Trial-by-trial error corrections were assessed by plotting the saccade correction on the next trial (ratio of the next primary-saccade amplitude to the current primary-saccade amplitude) against the saccade error on the current trial (absolute value of target endpoint minus the absolute value of saccade endpoint; all hypometric errors are expressed as positive values regardless of the direction of the primary saccade). Intervening corrective saccades, which bring the eyes to the target following a primary saccade, are not considered. Trends were measured by fitting the data with 95% confidence ellipses (CE_95_). The ellipse major axis describes the relationship between the error on the current trial and the correction on the next trial, and the minor axis describes scatter about that trend. The angle of the major axis describes how well errors are compensated on the next trial; angles closer to the ideal value of -3.01 radians (-172°) imply more complete compensation for errors (a 1° hypometric error for a 10° saccade should produce a gain correction of 1.11; since errors are small we approximate this hyperbolic relationship as a linear trend). The ideal tilt angle of -3.01 radians is also the angle that would result if each trial was independently drawn from a Gaussian distribution, centered at a gain of 1, prior to the error-correction analysis. This random process would yield the ideal compensation angle because it reflects the tendency for trials to be “corrected,” on average, toward the mean of the distribution (that is, to be more similar to the mean on a successive trial), which happens with high probability in a Gaussian distribution. Thus, the ideal error correction to bring the saccade gain back to the average saccade amplitude or the tendency of a Gaussian distribution to cluster about its mean yield the same effect in this analysis.

Surrogate data were used to test the possibility that observed trends simply arose by chance, as could happen if successive saccade amplitudes were simply random trials drawn from a given distribution. Each surrogate data set was generated by randomly shuffling the order of saccade amplitudes to destroy temporal correlations, then repeating the analysis of measuring endpoint errors and change in saccade gain between pairs of trials. Since this “whitens” the data or makes it random while still preserving the underlying amplitude distribution, it tends to produce confidence ellipses that are aligned closely with the ideal compensation angle of -3.01 radians.

Long-term correlations were quantified using two methods. The first analyzes the statistical correlation structure using the power spectrum. The power spectrum, *S_xx_*, is computed by taking the squared magnitude of the Fourier transform of the time series. Systems exhibiting long-term correlations (i.e., gradual decay) have power spectra that decay as a power-law; that is, for frequency *f*, 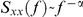. Thus, the negative slope of a linear regression of the power spectrum on a log-log plot provides an estimate of the scaling exponent α. Mathematically, such long-memory processes exhibit system dynamics on all time scales (no characteristic time scale), as suggested by the presence of significant fluctuations across all frequencies. On the other hand, state-space (ARMA) models exhibit faster (exponential) decay of inter-trial correlations and a more complex form of power spectrum. Evidence of power-law scaling, therefore, is particularly interesting because it suggests that the underlying time series is, statistically speaking, a process with long memory – longer than that exhibited by a state-space (ARMA) process [32].

To determine if a power-law is the best description for the power spectrum, these fits were compared against the alternative hypothesis that the power spectrum resulted from a simple state-space (ARMA) model of the form typically used to model motor learning [14]. State-space (ARMA) models exhibit exponential decay of inter-trial correlations, which can manifest as power spectra that resemble low-pass filters – in particular, they are flat in the low frequency range [33]. By approximating this as a piecewise-linear function with an inflection point, it was possible to test whether simple linear regressions or piecewise regressions best described the power spectra of the data. We allowed the fitting algorithm to select not only the slopes of the two halves of the piecewise regression, but also the inflection point. Goodness-of-fits were compared using the Bayesian Information Criterion (BIC), which accounts for not only the model residuals (via the computed log-likelihood of the model fit), but also the number of free parameters and the sample size. Smaller BIC values indicate better fits.

A modified bootstrap analysis was used to quantify how far into the past these “long-term” correlations extended [34]. To assess the minimum number of trials necessary to produce the observed long-memory, the time series was divided into sections of length Δ*N* and these sections were randomly shuffled, then recombined to form a new time series. By doing this, only fluctuations on time scales smaller than length Δ*N* are preserved. For example, for Δ*N* = 1, the shuffled time series approximates a white-noise process where each trial is independent, so this yields a value of α that is nearly zero. For each shuffled time series, α was computed. This process was repeated many times for each Δ*N* to yield an average estimate of the shuffled α value, which was then compared to the α value measured for the original data. The smallest Δ*N* for which α no longer differs statistically from that of the original data can be interpreted as the largest number of trials across which significant correlations exist in the data; that is, Δ*N* quantifies the extent of “long memory.”

The second technique for quantifying inter-trial correlations examines temporal scaling. The Hurst exponent (*H*) measures the extent to which the magnitude of a time series must be amplified to remain statistically identical as the time scale changes. *H* can be obtained using the “rescaled range” method [62]. The rescaled range is found by dividing the range, *R*, of an integrated time series by its standard deviation, *S*, for some duration *T* of the time series (i.e., the data are divided into segments of length *T*). Then, *H* is the slope on a log-log graph of the linear regression of the rescaled range versus *T*. The rescaled range is related to the length of a time series in a power law fashion, *R/S* 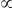 *T^H^*, for certain time series. *H* falls between zero and one; 0<*H*<0.5 indicates an anti-persistent process where large values tend to follow small values and vice versa, whereas 0.5<*H*<1 indicates a persistent process where large values tend to follow large values and small values follow small values. If *H* = 0.5, the process is completely random (white noise). Certain processes may have either anti-persistent or persistent trends present across multiple time scales, leading to the presence of long-term correlations [63]. The scaling exponents *H* and α are related by *H* = (1+α)/2 for α<1 (in the range of fractional Gaussian noise; see [64]). Thus, the computation of both exponents provides a means to check that the data are actually scale-invariant processes that exhibit long-range dependence. It has previously been demonstrated that certain types of dynamical processes can result in improper computation of either the *H* or α scaling exponent, so computing both independently allows us to verify these assessments of long-range dependence [31].

### The ARFIMA model, for fitting and simulation of time series with long memory

Modeling of time series that exhibit long-range dependence has been greatly advanced by the development of the Autoregressive Fractionally-Integrated Moving-Average (ARFIMA) process as an extension of the standard ARMA model [65] (for additional information, see Methods S1). An ARFIMA(*p*,*d*,*q*) process extends the ARMA(*p*,*q*) model by introducing a parameter *d* that describes how the time series is differenced prior to applying a conventional ARMA model; it accounts for long-term correlations. In other words, *d* reflects the order of any trend present in the data; for example, *d* = 1 represents a linear trend. Allowing *d* to take on fractional values – that is, by fractionally differencing the data prior to modeling [66] – the ARFIMA process can model long-range dependence. The value of *d* is related to the Hurst exponent by the relation *d* = *H* – 0.5; appropriate *d* values fall in the range (-0.5, 0.5). As with *H*, the value of *d* can indicate whether an ARFIMA model is persistent or anti-persistent. By comparing this value of *d* to values of *H* and α previously computed by independent methods, it is possible to verify that the measured scaling exponent is correct.

Data from an ARFIMA process were simulated using the Ox ARFIMA package [67]–[69]. ARFIMA and ARMA models were also fit to existing data using the same package. Model fits were compared using the Bayesian Information Criterion (BIC) [45], [70]. The BIC was used to assess whether ARMA models can capture the statistical characteristics and system dynamics present, or if a more complex ARFIMA model is better suited to describe the data.

## Supporting Information

Methods S1 **ARFIMA models.**
(DOC)
