# Supplementary material for: Exploring the Fundamental Dynamics of Error-Based Motor Learning Using a Stationary Predictive-Saccade Task
Source: PLoS One. 2011 Sep 23;6(9):e25225. doi: 10.1371/journal.pone.0025225 (PMC3179473; doi:10.1371/journal.pone.0025225)
Supplement: Methods S1 — ARFIMA models. (DOC) [file pone.0025225.s001.doc]

**Supplemental Methods: ARFIMA models**

Traditionally, motor learning has been modeled using state-space models, which describe learning as the updating of a state vector containing a discrete set of information regarding prior performance and current stimulus information. All state-space models have corresponding Autoregressive Moving-Average (ARMA) models [1]; that is, there exists some simple linear transformation to express a state-space model as an ARMA model [2]. This transformation is non-unique, such that a single ARMA model may be rewritten as a number of different state-space models. Nevertheless, the dynamics and statistics of these models are identical; both the state-space model and its ARMA counterpart will exhibit the same (exponential) decay of inter-trial correlations as expressed in the ACF and power spectrum.

An ARMA(*p*,*q*) model assumes that a time series may be described by two independent processes acting upon a white noise process. There is a *p*^th^ order autoregressive (AR) process that uses the previous *p* observations *X_t-p_*,…,*X_t-1_* and the current noise term *e_t_* to generate the next point in the time series *X_t_*, added to a *q*^th^ order moving-average (MA) process that uses the last *q* noise terms *e_t-q_*,…,*e_t-1_* to generate the next observation. This yields an ARMA(*p*,*q*) process of the form:

$X_{t}=\sum_{j=1}^{p} \varphi_{j}X_{t-j}+e_{t}+\sum_{k=1}^{q} \theta_{k}e_{t-k}$ (Eqn. 1)

where the $\varphi_{j}$ and the $\theta_{k}$are constant coefficients. An ARMA process only models short-term correlations in a time series, limited by the extent over which prior observations and noise terms are explicitly incorporated. To exhibit inter-trial correlations that decay more slowly than an exponential rate, it is necessary to turn to a different class of models.

The ARFIMA(*p*,*d*,*q*) model is a combination of the standard ARMA(*p*,*q*) model [1], which describes a process exhibiting short-term correlations, and a fractional differencing parameter, *d*, which gives the model its long-range dependence. Eqn. 1 can be re-written using the backward shift operator *B*, defined as *BX_t_* = *X_t_*_-1_, as a polynomial in *B* with coefficients $\varphi_{j}$ and the $\theta_{k}$:

$\varphi\left(B\right)X_{t}=\theta\left(B\right)e_{t}$, (Eqn. 2)

where powers of *B* indicate a corresponding number of shifts backward in the time series. Note that using *B*, we can express the differencing of successive terms in the time series as (1-*B*)*X_t_*; raising (1-*B*) to the *d*^th^ power is equivalent to repeatedly differencing the time series *d* times. If *d* is a fraction in the range 0 < $\left|d\right|$ ≤ 0.5, we then have the fractional differencing operator defined using the binomial expansion by:

$\left(1-B\right)^{d}=\sum_{k=0}^{\infty} \left(\frac{\Gamma\left(d+1\right)}{\Gamma\left(k+1\right)\Gamma\left(d-k+1\right)}\right)\left(-B\right)^{k}$, (Eqn. 3)

where Γ is the gamma function [3-4]. Thus, we can express an ARFIMA model as:

$\left(1-B\right)^{d}\varphi\left(B\right)X_{t}=\theta\left(B\right)e_{t}$. (Eqn. 4)

The fractional integration gives the model its long-range dependence [5]; thus, an ARFIMA model exhibits the expected power-law type decay of the autocorrelation function that marks it as having long-term correlations. ARFIMA models with *d* values greater than zero are persistent – small values tend to be followed by small values and large values tend to be followed by large values – while *d* values less than zero produce anti-persistent processes that tend to fluctuate highly. This model provides a neat and compact form for expressing the complexities of a process exhibiting long-range dependence. Since fractional integration requires keeping track of all data points in a time series to an infinite time in the past, however, the computations for simulating or modeling an ARFIMA process can be quite unwieldy.

**References**

1. Box GEP, Jenkins GM (1970) Time series analysis: forecasting and control. Oakland: Holden-Day.

2. Akaike H (1974) Markovian representation of stochastic proceses and its application to analysis of autoregressive moving average processes. Ann Inst Statist Math 26: 363-387.

3. Hosking JRM (1981) Fractional Differencing. Biometrika 68: 165-176.

4. Beran J (1994) Statistics for long-memory processes. New York: Chapman & Hall.

5. Granger CWJ, Joyeux R (1980) An introduction to long-range time series models and fractional differencing. J Time Series Analysis 1: 15-29.
